# Prognostic value of programmed cell death ligand 1 expression in patients with intrahepatic cholangiocarcinoma: a meta-analysis

**DOI:** 10.3389/fimmu.2023.1119168

**Published:** 2023-04-17

**Authors:** Feng Xian, Dacheng Ren, Jun Bie, Guohui Xu

**Affiliations:** ^1^ School of Medicine, University of Electronic Science and Technology of China, Chengdu, China; ^2^ Department of Oncology, Nanchong Central Hospital, the Second Clinical Medical College, North Sichuan Medical College, Nanchong, China; ^3^ Department of Interventional Radiology, Sichuan Clinical Research Center for Cancer, Sichuan Cancer Hospital & Institute, Sichuan Cancer Center, Affiliated Cancer Hospital of University of Electronic Science and Technology of China, Chengdu, China

**Keywords:** intrahepatic carcinoma, prognostic value, meta-analysis, programmed cell death ligand 1 (PDL1), programmed cell death 1 (PD-1)

## Abstract

**Background:**

Programmed cell death ligand 1 (PD-L1) is highly expressed in intrahepatic cholangiocarcinoma (ICC) tissues. But there is still a dispute over the prognostic value of PD-L1 in patients with ICC. This study aimed to evaluate the prognostic value of PD-L1 expression in patients with ICC.

**Methods:**

We performed a meta-analysis based on the Preferred Reporting Items for Systematic Reviews and Meta-Analyses Guidelines. We searched the literature from PubMed, Embase, Web of Science, and the Cochrane Library up to December 5, 2022. Hazard ratios (HR) and their 95% confidence intervals (95% CI) were calculated to analyze the overall survival (OS), recurrence-free survival (RFS), and time to relapse. The quality of the studies was assessed using the Newcastle-Ottawa scale. Publication bias was assessed using a funnel plot and Egger’s test.

**Results:**

Ten trials with 1944 cases were included in this meta-analysis. The results showed that the low-PD-L1 group had a statistically significant advantage in OS (HR, 1.57; 95% CI, 1.38–1.79, P <0.00001), RFS (HR, 1.62; 95% CI, 1.34–1.97, P <0.00001), and time to relapse (HR, 1.60; 95% CI, 1.25–2.05, P = 0.0002) compared with the high-PD-L1 group. High programmed cell death (PD1)levels, on the other hand, were correlated with poorer OS (HR, 1.96; 95% CI, 1.43–2.70; P <0.0001) and RFS (HR, 1.87; 95% CI, 1.21–2.91; P = 0.005). Multivariate analysis showed that PD-L1 could act as an independent predictor for OS (HR, 1.48; 95% CI, 1.14–1.91; P = 0.003) and RFS (HR, 1.74; 95% CI, 1.22–2.47; P = 0.002), and PD1 acted as an independent predictor for OS (HR, 1.66; 95% CI, 1.15–2.38; P = 0.006).

**Conclusion:**

This meta-analysis demonstrated that high PD-L1/PD1 expression is associated with poor survival in ICC. PD-L1/PD1 may be a valuable prognostic and predictive biomarker and potential therapeutic target in ICC.

**Systematic review registration:**

https://www.crd.york.ac.uk/PROSPERO/, identifier CRD42022380093.

## 1 Introduction

Intrahepatic cholangiocarcinoma (ICC) accounts for over 20% of primary liver cancers and is the second most common primary liver tumor after hepatocellular carcinoma (1). ICC is a highly lethal neoplasm because it is always misdiagnosed and there is no effective therapeutic regimen to control it, which is not sensitive to chemotherapy and radiotherapy (2). Therefore, an effective therapeutic target has recently been focused on ICC worldwide.

The programmed cell death (PD) ligand 1 (PD1)/PD-L1 axis is one of the most important immune checkpoints and a valuable therapeutic target because it plays a critical role in facilitating immune evasion (3). Tumor cells express PD-L1, which binds to PD1 to evade antitumor responses (4). PD-L1 also plays an important role in subsequent tumor progression by binding to PD1 and activating proliferative and survival signaling pathways (5). Furthermore, PD-L1 expression can be modulated by various tumorigenesis-related signaling pathways, including the phosphoinositide 3-kinase–protein kinase B/Akt (PI3K/AKT) (6–8), mitogen-activated protein kinase (MAPK) (9), and Wingless/Integrated (WNT) pathways (10). An increasing number of studies have clearly demonstrated that PD-L1 is overexpressed in various malignant tumors, such as nasopharyngeal carcinoma, lung cancer, and hepatocellular carcinoma (11–16). Additionally, PD-L1 expression is significantly associated with prognosis and clinicopathological features; for example, PD-L1 overexpression contributes to poor prognosis in gastric, colorectal, breast, and pancreatic cancers, and renal cell and hepatocellular carcinomas (17–21). Furthermore, many studies have found that PD-L1 is highly expressed in ICC and is markedly related to the prognosis of patients with ICC (22, 23). However, few studies have reported the efficacy of anti-PD1/PD-L1 treatment for ICC (24, 25), and PD-L1 as a therapeutic target for ICC is still in demand, which requires further evaluation.

Therefore, we performed this meta-analysis to assess the effect of PD-L1 expression on the prognosis of ICC. The purpose of this study was to evaluate the correlation between PD-L1 overexpression and survival in ICC, thereby shedding more light on the development of PD-L1 targeted therapy and prognostic prediction.

## 2 Materials and methods

This study has been conducted in accordance with the Preferred Reporting Items for Systematic Reviews and Meta-Analyses Guidelines (26, 27) (**Supplementary Table 1**).

### 2.1 Search strategy

Two investigators independently searched the online databases PubMed, Embase, Cochrane Library, and Web of Science for interrelated studies up to December 5, 2022. The search terms were “intrahepatic cholangiocarcinoma” AND “programmed cell death ligand 1” OR “PD-L1” OR “B7-H1” (**Supplementary Table 2**). Reference lists from published studies were also searched.

### 2.2 Inclusion and exclusion criteria

The inclusion criteria were as follows: (1) patients who were diagnosed with ICC; (2) articles that were published in English with full texts, involving humans as study subjects; (3) the correlation between PD-L1 and survival (overall survival [OS], recurrence-free survival [RFS], and time to relapse [TTR]) was detected; (4) the hazard ratio (HR) and 95% confidence intervals (CI) for survival times were computed by included articles that provided sufficient data; and (5) PD-L1 expression levels were gauged in clinical ICC tissues. The exclusion criteria were as follows: (1) patients with ICC and other cancers; (2) case reports, abstracts, and reviews; (3) duplicated articles; and (4) articles with incomplete data.

### 2.3 Data extraction

Two reviewers independently extracted the data. Any disagreements regarding the information were resolved by consensus or by the judgment of a third reviewer. The following data were extracted from each study: authors of the article, year of publication, detection method, region, cutoff value, follow-up time, and HRs with 95% CI (28). The main outcomes were PD-L1 expression-related OS, RFS, and TTR. The following data were also extracted if the study contains: PD1 expression-related survival data. While the original survival data were hardly accessed, the extracted data from the Kaplan–Meier curves were obtained using the software Engauge Digitizer version12.1.

### 2.4 Quality assessment

Two reviewers independently evaluated the quality of the included literature using a 9-score system of the Newcastle-Ottawa Scale (NOS) (29). The NOS comprises eight items, with a score >6 indicating high-quality research.

### 2.5 Statistical analysis

All data analyses were performed using Review Manager version 5.3 and Stata Software version 14.0. We applied pooled HRs with their 95% CIs to evaluate the association between prognostic value and the expression levels of PD-L1 in ICC. Cochran’s Q and I^2^ statistics were used to assess the heterogeneity among these articles (30). Heterogeneity was considered insignificant when P >0.10 or I^2^ <50%, and a fixed-effects model was used to pool the effects; otherwise, a random-effects model was used. Funnel plot and Egger’s test were used to estimate publication bias, and a P value of <0.05 implied statistical significance.

## 3 Results

### 3.1 Selected studies

**Figure 1:**
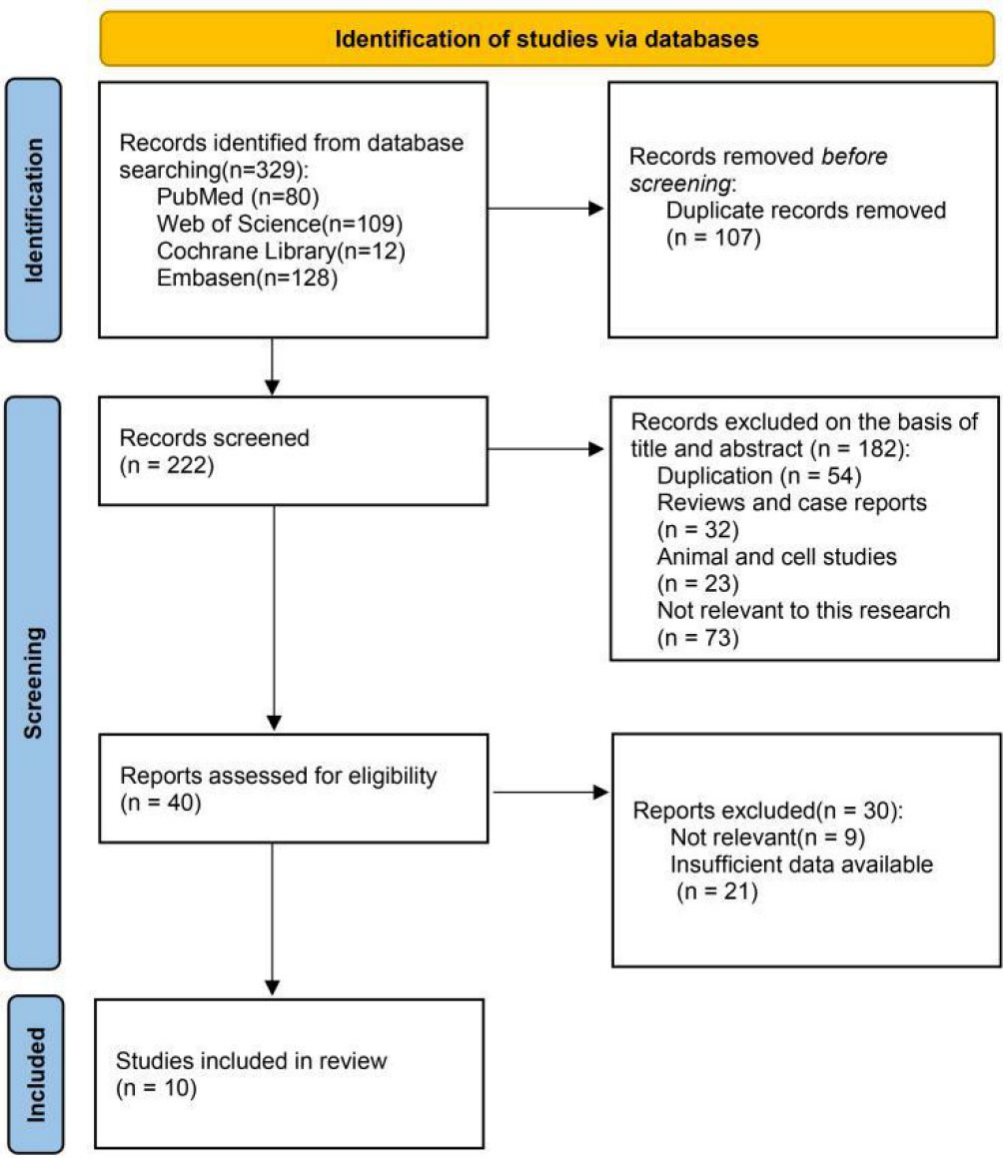
Flow diagram of study selection.

We searched for 329 trials in four electronic databases. Subsequently, we excluded 107 duplicates, 182 records after screening the titles and abstracts, and 30 records due to irrelevant or incomplete data. Eventually, ten trials (22, 31–39) and 1944 cases were included according to the inclusion and exclusion criteria (**Figure 1**).

### 3.2 Study characteristics and quality assessment

Nine studies were included from Asia (China, Korea, and Japan), and one was from Germany. All of the included studies used either immunohistochemistry or immunofluorescence to detect PD-L1 expression. Moreover, we extracted two sets of survival data from one trial with the training and validation cohorts. Four studies reported the effect of PD1 on the prognosis of ICC. The quality of the included trials was assessed by NOS, and all included studies were high-quality studies. The NOS scores and characteristics of the included studies are summarized in **Table 1**, the quality evaluations of all included articles are in **Supplementary Table 3**.

**Table 1:** The characteristics of the included studies.

Author	Year	Country	Type of study	Case number (High/Low)	Cut-off	Scoring method	Method	PD-L1 or PD1	Outcome	Follow-up (years)	NOS Scores
Ha et al. (31)	2016	Korea	Retrospective	158 (58/100)	5%	H-score	IHC	PD-L1	OS	9.8 (median)	8
Lan et al. (32)	2022	Japan	Retrospective	158 (NR/NR)	90%	H-score	IHC	PD-L1	OS	2.9 (median)	8
Lu et al. (22)	2019	China	Retrospective	320 (99/221)	5%(PD-L1) median(PD1)	H-score	IHC	PD-L1 and PD1	OS, RFS	11 (maximum)	8
Tan et al. (33)	2022	Germany	Retrospective	45 (15/30)	1.4x10^-6^	IPS	IF	PD-1	OS, RFS	NR	7
Tao et al. (training) (34)	2020	China	Retrospective	214 (107/107)	55%	H-score	IHC	PD-L1	OS, TTR, RFS	5 (maximum)	8
Tao et al. (validation) (34)	2020	China	Retrospective	108 (54/54)	55%	H-score	IHC	PD-L1	OS, TTR, RFS	5 (maximum)	8
Tian et al. (35)	2020	China	Retrospective	322 (80/242)	NR	NR	IHC	PD-L1 and PD1	OS	2.25 (median)	8
Yang et al. (36)	2022	China	Retrospective	81 (46/35)	median	H-score	IHC	PD1	OS, RFS	5 (maximum)	8
Yugawa et al. (37)	2021	Japan	Retrospective	100 (49/51)	1%	H-score	IHC	PD-L1	OS, RFS	NR	7
Zheng et al. (38)	2022	China	Retrospective	99 (18/81)	2.56%	H-score	IHC	PD-L1	RFS	10 (maximum)	8
Kim et al. (39)	2021	Korea	Retrospective	72 (18/54)	1	CPS	IHC	PD-L1	OS	NR	7

NR, not reported; IHC, immunohistochemistry; ELISA, enzyme-linked immunosorbent assay; IPS, immune cell proportion score; CRS, combined positive score; PD-L1, programmed cell death ligand 1; OS, overall survival; RFS, recurrence-free survival; TTR, time to relapse; NOS, Newcastle-Ottawa Scale.

### 3.3 OS

**Figure 2:**
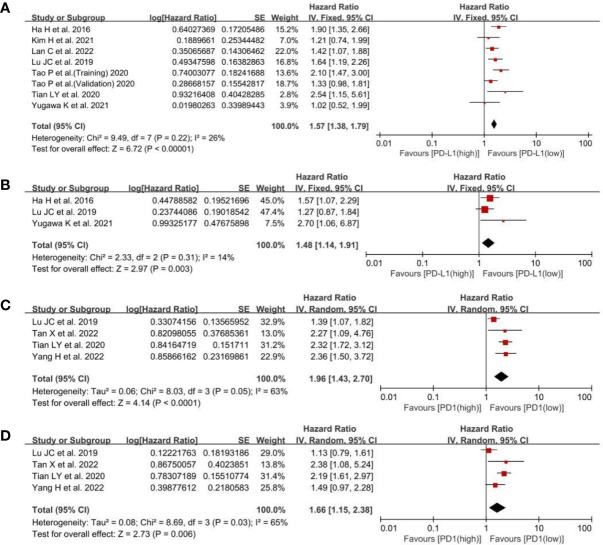
A forest plot of OS between low- and high-PD-L1 expression: **(A)** pooled OS by univariate analysis; **(B)** pooled OS by multivariate analysis. A forest plot of OS between low- and high-PD1 expression: **(C)** pooled OS by univariate analysis; **(D)** pooled OS by multivariate analysis.PD-L1, programmed cell death ligand 1; OS, overall survival; SE, standard error.

Regarding the effect of PD-L1 expression on OS, the low-PD-L1 group had a significantly better OS (HR, 1.57; 95% CI, 1.38–1.79; P <0.00001; I^2^, 26%; P = 0.22) than the high-PD-L1 group (**Figure 2A**). Furthermore, multivariate analysis showed that PD-L1 was also a significant independent prognostic factor (HR, 1.48; 95% CI, 1.14–1.91; P = 0.003; I^2^, 14%; P = 0.31) (**Figure 2B**). While concerning the effect of PD1 expression on OS, a statistically significant difference in OS (HR, 1.96; 95% CI, 1.43–2.70; P <0.0001) was found between the PD1 low and high groups (**Figure 2C**). Multivariate analysis showed that PD1 was also a significant independent prognostic factor (HR, 1.66; 95% CI, 1.15–2.38; P = 0.006) (**Figure 2D**). Namely, both high PD-L1 and PD1 expression were associated with poor OS of patients with ICC.

### 3.4 RFS

**Figure 3:**
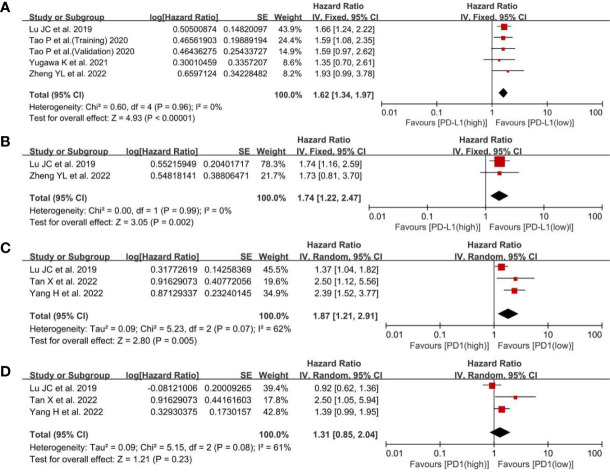
A forest plot of RFS between low- and high-PD-L1 expression: **(A)** pooled RFS in univariate analysis; **(B)** pooled RFS in multivariate analysis. A forest plot of RFS between low- and high-PD1 expression: **(C)** pooled RFS in univariate analysis; **(D)** pooled RFS in multivariate analysis. RFS, recurrence-free survival; PD-L1, programmed cell death ligand 1; SE, standard error.

Regarding the effect of PD-L1 expression on RFS, the low-PD-L1 group had a statistically significant advantage in RFS (HR, 1.62; 95% CI, 1.34–1.97; P <0.00001) in univariate analysis and (HR, 1.74; 95% CI, 1.22–2.47; P = 0.002) (**Figures 3A, B**), means that the high level of PD-L1 was relevant to poor RFS and may be used to predict the recurrence time after antitumor therapy. For the effect of PD1 expression on RFS, there was a statistically significant difference in RFS (HR, 1.87; 95% CI, 1.21–2.91; P = 0.005) (**Figure 3C**), whereas multivariate analysis did not show any statistically significant difference in RFS (HR, 1.31; 95% CI, 0.85–2.04; P = 0.23; **Figure 3D**). The HRs of the RFS for PD1 expression were pooled using a random-effects model because there was slight heterogeneity among the three studies (I^2^, 62%; P = 0.07). Based on the above, the PD1 overexpression could be related to poor RFS, but there were not any correlations between PD1 levels and RFS in multivariate analysis.

### 3.5 TTR

**Figure 4:**
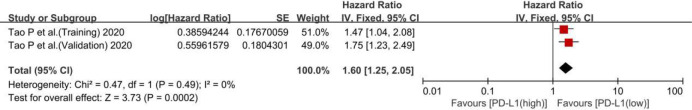
A forest plot of time to relapse between low- and high-PD-L1 expression. PD-L1, programmed cell death ligand 1; SE, standard error.

Two cohorts in one trial reported the TTR of patients with PD-L1 expression. The results showed that the low-PD-L1 group had a statistically significant advantage in TTR (HR, 1.60; 95% CI, 1.25–2.05; P = 0.0002; I^2^, 0%; P = 0.49) (**Figure 4**). So, the low level of PD-L1 was beneficial to well TTR of ICC patients.

### 3.6 Subgroup analysis for PD-L1 expression

**Figure 5:**
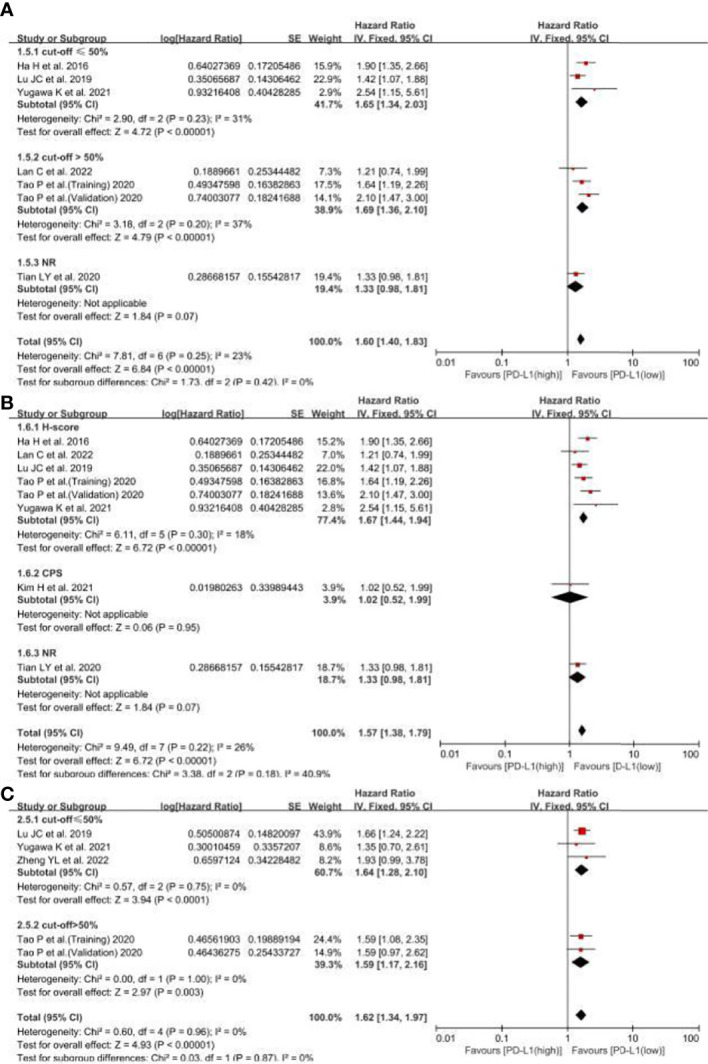
The subgroup analyses for PD-L1 expression related OS and RFS. **(A)** the subgroup analyses for PD-L1 expression related OS based on different cut-offs, **(B)** the subgroup analyses for PD-L1 expression related OS based on different scoring methods, **(C)** the subgroup analyses for PD-L1 expression related RFS based on cut-offs. NR, not reported.

Considering that cut-offs for PD-L1 positivity, the scoring method may be important sources of heterogeneity, we conducted the subgroup analyses based on the cut-offs and scoring methods. The subgroups were divided by cut-offs (≤50%, >50% and not reported) and scoring methods (IHC, CPS and not reported). In OS, the results showed there were not any differences among the cut-off groups (I^2^, 0%; P = 0.42) (**Figure 5A**), and the scoring method groups (I^2^, 40.9%; P = 0.18) (**Figure 5B**). Besides, the cut-off groups also had no differences (I^2^, 0%; P = 0.87) (**Figure 5C**). Therefore, the cut-offs and scoring methods may not be the important sources of heterogeneity, means the different cut-offs and scoring method could not affect the pooled HRs.

### 3.7 Sensitivity analysis

**Figure 6:**
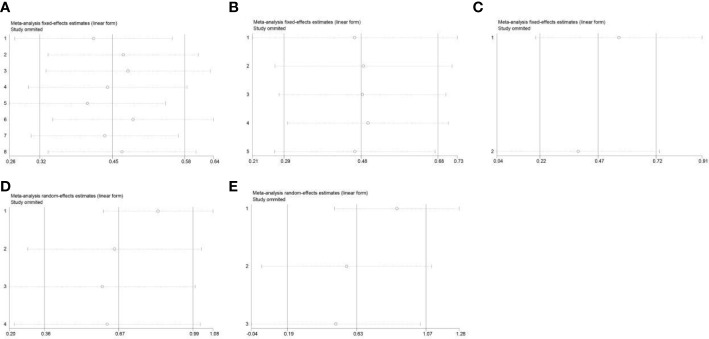
Sensitivity analysis of the meta-analysis: **(A)** OS in PD-L1 expression; **(B)** RFS in PD-L1 expression; **(C)** TTR in PD-L1 expression; **(D)** OS in PD1 expression; **(E)** RFS in PD1 expression. PD-L1, programmed cell death ligand 1; OS, overall survival; RFS, recurrence-free survival.

We conducted a sensitivity analysis for OS (**Figure 6A**), RFS (**Figure 6B**), and TTR (**Figure 6C**) for PD-L1 expression and OS (**Figure 6D**) and RFS (**Figure 6E**) for PD1 expression in ICC by separately removing each study and then merging the effect quality. After excluding each study from the main analysis, the overall results showed no significant changes, which suggests that our combined results are reliable.

### 3.8 Publication bias

**Figure 7:**
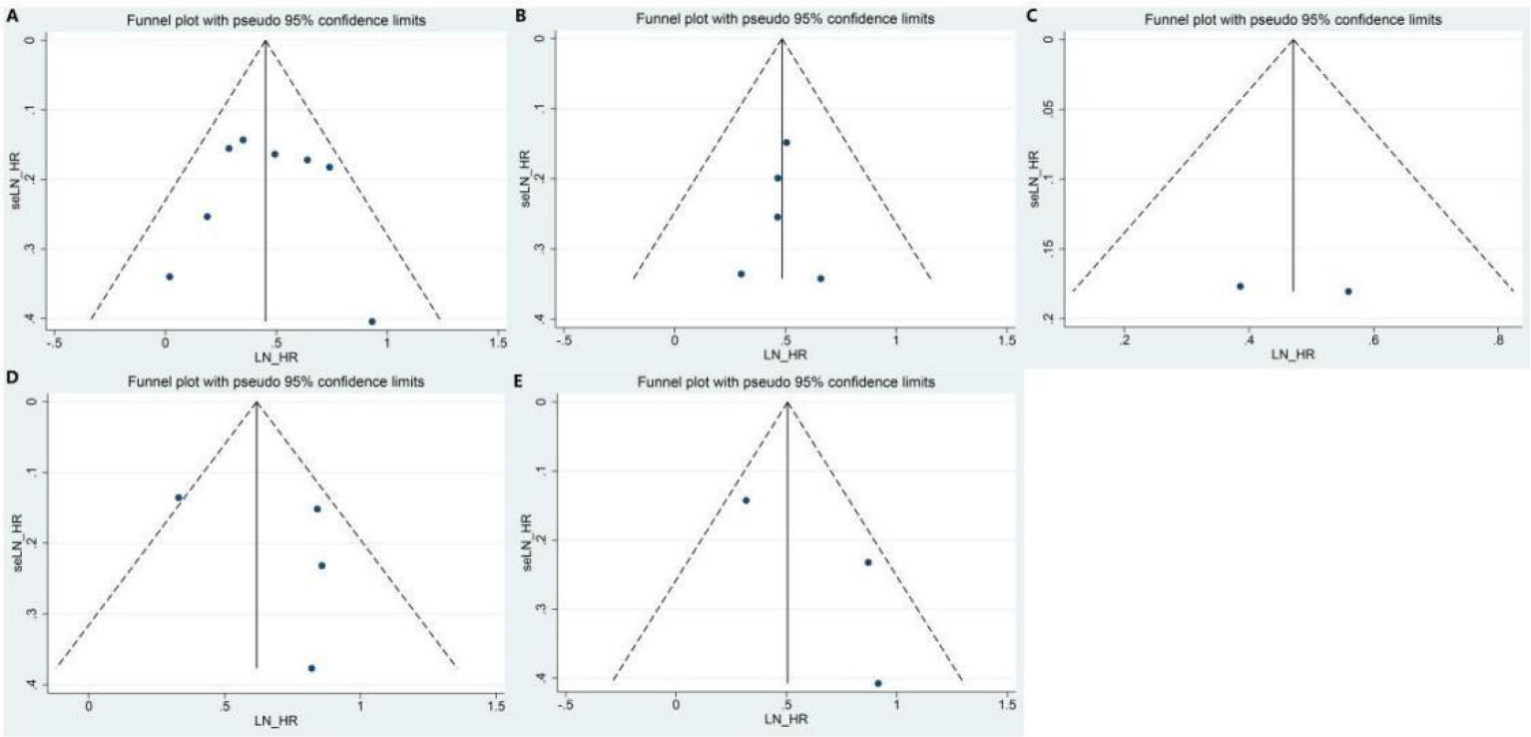
Funnel plot of the meta-analysis: **(A)** OS in PD-L1 expression; **(B)** RFS in PD-L1 expression; **(C)** TTR in PD-L1 expression; **(D)** OS in PD1 expression; **(E)** RFS in PD1 expression. PD-L1, programmed cell death ligand 1; OS, overall survival; RFS, recurrence-free survival; TTR, time to relapse.

The funnel plots and Egger’s test were used to test for potential publication bias. The funnel plot showed that the left and right sides were symmetrical (**Figures 7A–E**). Egger’s test showed that all P >0.1, indicating a low probability of publication bias.

## 4 Discussion

The high recurrence rate after surgical removal and low disease control response to treatment for ICC have become a problem for doctors and patients. This study is the first meta-analysis of the prognostic value of PD-L1 expression in patients with ICC. The results showed the prognostic value of PD-L1/PD1 staining in ICC.

In our study, the OS of the low-PD-L1 group was 1.57 times longer than that of the high group. This finding indicates that the prognosis of patients with high PD-L1 levels is worse due to the ability of PD-L1 to facilitate immune evasion (3). Interferon-γ can induce protein kinase D isoform, which can cause PD-L1 upregulation in various solid cancers, and inhibiting its activity could suppress PD-L1 expression and promote an obvious antitumor immune response (40). Moreover, the multivariate analysis also showed that the OS of the low-PD-L1 group was significantly different (HR, 1.96; P<0.0001), indicating that PD-L1 expression was an independent indicator of prognosis in patients with ICC. This finding is consistent with the feature of PD-L1 as a pro-tumorigenic factor (5).

The low-PD-L1 group also had statistically significant advantages in RFS and TTR compared to the high-PD-L1 group (HR, 1.74; P <0.00001 vs. HR, 1.60; P = 0.0002, respectively). These findings confirmed that patients with high PD-L1 expression had significantly higher recurrence rates than those with low PD-L1, especially after curative resection. Multivariate analysis showed that the RFS of the low-PD-L1 group was 1.74 times longer than that of the high-PD-L1 group. This indicates that PD-L1 levels could act as independent predictors of RFS and elevated recurrence. These results may be correlated with the fact that PD-L1 is active and maintains the proliferation of tumor cells (5). For example, the intrinsic pathway of PD-L1 promotes renal cell carcinoma progression by inducing stem cell-like phenotypes in renal cancer cells (41).

Furthermore, we analyzed the effect of PD1 expression on the prognosis of ICC. The results showed that the OS and RFS of PD1 low levels were statistically significantly longer than those of high levels (HR, 1.66; P = 0.006 vs. HR, 1.87; P = 0.005, respectively), which illustrated that PD1 has potential prognostic value for ICC. Multivariate analysis showed that PD1 also acted as an independent predictor for the OS of ICC (HR, 1.87; P = 0.005). PD1 is mainly expressed in activated immune cells as an inhibitor of innate immune responses. PD1 regulates the immune microenvironment by binding to PD-L1; therefore, in theory, PD1 has a similar predictive effect to PD-L1 (42). However, no statistically significant difference in RFS was observed in the multivariate analysis (HR, 1.31; P = 0.23), indicating that PD1 was not an independent predictor of RFS. Which is different from the relationship between PD1 and RFS in other cancers, such as PD1 positivity was an independent predictor of RFS in renal cell carcinoma (43). The number of infiltrated immune cells and the limited number of trials that were included could change the results. The correlation between PD1 expression and the RFS of ICC need to be further explored in future studies.

Therefore, PD-L1/PD1 inhibitors can be used as effective target medications for patients with ICC who exhibit PD-L1/PD1 overexpression. Zhu et al. reported that stage IIIB ICC could be treated with neoadjuvant therapy with a PD1 inhibitor (44). PD-L1/PD1 inhibitors combined with targeted therapy, chemotherapy, and radiotherapy had a synergistic effect on ICC, which improved disease control responses (24, 45, 46). PD-L1/PD1 inhibitors have achieved good results in the treatment of advanced or neoadjuvant ICC. We expect that this regimen could be utilized for patients with ICC as a first-line treatment rather than a salvage treatment.

In order to exclude other factors, we conducted subgroup analyses based on cut-offs and scoring methods. The results showed that both them did not change the pooled results, which is similar to some studies (47). For example, good overall concordance on analytic performance was observed for two assays (Dako 22C3 and Ventana SP263) with both scoring algorithms: the combined positive score (CPS) and tumor infiltrating immune cells (IC), but the SP 142 showed lower positivity rates, especially using the CPS algorithm (48). So different scoring methods could still affect the results. Besides, there were a similar correlation between PD-L1 levels and survival in papillary thyroid carcinoma, PD-L1 expression was significantly associated with a reduced disease-free survival (49). However, no association was found with the OS (49), which is opposite to the results in ICC. Which may be related to the different intensity of promoting cancer progression of PD-L1 in different cancers. Based on the above, the influence of PD-L1 expression on tumor prognosis may vary with different IHC assays, scoring methods and tumor types.

Our study has several limitations. First, due to a lack of data, further subgroup analysis according to clinicopathological features was not conducted. Second, because the majority of patients in our study were from Asia, the results could not fully represent other groups worldwide. Finally, the sample sizes of the included studies were insufficient to conduct a good pooled analysis, especially for PD1. Therefore, we expect that more valuable related trials will be reported in the future.

## 5 Conclusion

The meta-analysis suggested that high-PD-L1 expression levels predicted poorer OS, RFS, and TTR in patients with ICC. PD1 expression levels are also related to the prognosis of patients with ICC. Moreover, both PD-L1 and PD1 were independent predictors of OS, and PD-L1 was an independent predictor of RFS. Our results indicate that PD-L1/PD1 has great potential as an effective prognostic biomarker and therapeutic target in ICC.

## Data availability statement

The raw data supporting the conclusions of this article will be made available by the authors, without undue reservation.

## Author contributions

FX and GX designed the study. FX and DR screened the studies and extracted data. The quality of the evidence was assessed using FX and JB. FX and DR analyzed and interpreted the data. FX and DR prepared figures and drafted the manuscript. JB and GX contributed to reviewing and editing the manuscript. All authors have approved the final version of the article, including the authorship list. All authors contributed to the article.
