# Supplementary material for: Prognostic value of programmed cell death ligand 1 expression in patients with intrahepatic cholangiocarcinoma: a meta-analysis
Source: Front Immunol. 2023 Apr 17;14:1119168. doi: 10.3389/fimmu.2023.1119168 (PMC10149806; doi:10.3389/fimmu.2023.1119168)
Supplement: Supplementary file 2 [file Table_2.docx]

**Supplementary Table 2** The search strategy for each database.

| **Database** | **Search strategy** |
| --- | --- |
| PubMed | **((Cholangiocarcinoma, Intrahepatic[Title/Abstract]) OR (intrahepatic cholangiocarcinoma[Title/Abstract])) AND (((((((PD-L1[Title/Abstract]) OR (B7-H1[Title/Abstract])) OR (B7H1[Title/Abstract])) OR (programmed cell death ligand 1[Title/Abstract])) OR (CD274[Title/Abstract])) OR (PDL1[Title/Abstract])) OR (programmed cell death 1 Ligand 1[Title/Abstract]))** |
| Web of Science | **(TS=(intrahepatic cholangiocarcinoma OR Cholangiocarcinoma, Intrahepatic)) AND TS=(PD-L1 OR B7-H1 OR B7H1 OR programmed cell death ligand 1 OR CD274 OR PDL1 OR programmed cell death 1 Ligand 1)** |
| Cochrane Library | **#1. (intrahepatic cholangiocarcinoma):ab,ti,kw OR (Cholangiocarcinoma, Intrahepatic):ab,ti,kw** |
|  | **#2. PD-L1):ab,ti,kw OR (B7H1):ab,ti,kw OR (programmed cell death ligand 1):ab,ti,kw OR (CD274):ab,ti,kw OR (PDL1):ab,ti,kw OR (programmed cell death 1 Ligand 1):ab,ti,kw** |
|  | **#3. #1 AND #2** |
| Embase | **#1. 'pd-l1':ab,ti OR 'b7-h1':ab,ti OR 'b7h1':ab,ti OR 'programmed cell death ligand 1':ab,ti OR 'cd274':ab,ti OR 'pdl1':ab,ti OR 'programmed cell death 1 ligand 1':ab,ti** |
|  | **#2. 'intrahepatic cholangiocarcinoma':ab,ti OR 'cholangiocarcinoma, intrahepatic':ab,ti** |
|  | **#3. #1 AND #2** |
