# Supplementary material for: Prognostic value of programmed cell death ligand 1 expression in patients with intrahepatic cholangiocarcinoma: a meta-analysis
Source: Front Immunol. 2023 Apr 17;14:1119168. doi: 10.3389/fimmu.2023.1119168 (PMC10149806; doi:10.3389/fimmu.2023.1119168)
Supplement: Supplementary file 3 [file Table_3.docx]

**Supplementary Table 3** The quality evaluations of all included articles.

| **Author** | **Year** |  | **Selection** | | | |  | **Comparability** | |  | **Outcome** | | |  | **Total score** |
| --- | --- | --- | --- | --- | --- | --- | --- | --- | --- | --- | --- | --- | --- | --- | --- |
|  |  |  | **(1)** | **(2)** | **(3)** | **(4)** |  | **(1)** | **(2)** |  | **(1)** | **(2)** | **(3)** |  |  |
| Ha et al. [31] | 2016 |  | **🟑** | **🟑** | **🟑** | - |  | **🟑** | **🟑** |  | **🟑** | **🟑** | **🟑** |  | 8 |
| Lan et al. [32] | 2022 |  | **🟑** | **🟑** | **🟑** | - |  | **🟑** | **🟑** |  | **🟑** | **🟑** | **🟑** |  | 8 |
| Lu et al. [33] | 2019 |  | **🟑** | **🟑** | **🟑** | - |  | **🟑** | **🟑** |  | **🟑** | **🟑** | **🟑** |  | 8 |
| Tan et al. [34] | 2022 |  | **🟑** | **🟑** | **🟑** | - |  | **🟑** | - |  | **🟑** | **🟑** | **🟑** |  | 7 |
| Tao et al. [35] | 2020 |  | **🟑** | **🟑** | **🟑** | - |  | **🟑** | **🟑** |  | **🟑** | **🟑** | **🟑** |  | 8 |
| Tian et al. [36] | 2020 |  | **🟑** | **🟑** | **🟑** | - |  | **🟑** | **🟑** |  | **🟑** | **🟑** | **🟑** |  | 8 |
| Yang et al. [37] | 2022 |  | **🟑** | **🟑** | **🟑** | - |  | **🟑** | **🟑** |  | **🟑** | **🟑** | **🟑** |  | 8 |
| Yugawa et al. [38] | 2021 |  | **🟑** | **🟑** | **🟑** | - |  | **🟑** | - |  | **🟑** | **🟑** | **🟑** |  | 7 |
| Zheng et al. [39] | 2022 |  | **🟑** | **🟑** | **🟑** | - |  | **🟑** | **🟑** |  | **🟑** | **🟑** | **🟑** |  | 8 |
| Kim et al. [40] | 2021 |  | **🟑** | **🟑** | **🟑** | - |  | **🟑** | - |  | **🟑** | **🟑** | **🟑** |  | 7 |

**Selection 0-4🟑**

(1) Representativeness of the exposed cohort

(2) Selection of the non exposed cohort

(3) Ascertainment of exposure

(4) Demonstration that outcome of interest was not present at start of study

**Comparability 0-2🟑**

Comparability of cohorts on the basis of the design or analysis

(1) Study controls for _____________ (select the most important factor)

(2) Study controls for any additional factor (This criteria could be modified to indicate specific control for a second important factor.)

**Outcome 0-3🟑**

(1) Assessment of outcome

(2) Was follow-up long enough for outcomes to occur

(3) Adequacy of follow up of cohorts
